# First record of the genus *Conotalopia* Iredale, 1929 (Vetigastropoda, Trochidae) in China

**DOI:** 10.3897/BDJ.12.e117114

**Published:** 2024-06-11

**Authors:** Lu Qi, Biyang Xu, Lingfeng Kong, Qi Li

**Affiliations:** 1 Key Laboratory of Mariculture, Ministry of Education, Ocean University of China, Qingdao, China Key Laboratory of Mariculture, Ministry of Education, Ocean University of China Qingdao China; 2 Institute of Marine Science and Technology, Shandong University, Qingdao, China Institute of Marine Science and Technology, Shandong University Qingdao China; 3 Sanya Oceanographic Institution, Ocean University of China, Sanya, China Sanya Oceanographic Institution, Ocean University of China Sanya China; 4 Laboratory for Marine Fisheries Science and Food Production Processes, Laoshan Laboratory, Qingdao, China Laboratory for Marine Fisheries Science and Food Production Processes, Laoshan Laboratory Qingdao China

**Keywords:** micromolluscs, new record, morphology, systematics

## Abstract

**Background:**

The genus *Conotalopia* Iredale, 1929 consisting of marine trochids, primarily inhabits the intertidal zone. Globally, eight recent species have been documented, all of which occur in the Pacific Region. The genus has not previously been recorded from Chinese seas.

**New information:**

This study fills a knowledge gap by reporting, for the first time, the presence of the trochid genus *Conotalopia* Iredale, 1929 in China. Specifically, *Conotalopiasematensis* (Oyama, 1942) was detailed using morphological characteristics derived from the shell (Fig. 1A-F and H-I), operculum (Fig. 1G) and radula (Fig. 1J-L). Additionally, this study introduces comprehensive scanning electron microscope illustrations and molecular data, contributing valuable taxonomic information for the first time.

## Introduction

The genus *Conotalopia* Iredale, 1929 represents a small group of marine trochids (Gastropoda, Trochidae), that are found in the intertidal zone and associated with algal vegetation. The type species of *Conotalopia* is *Monileahenniana* (Melvill, 1891) by its original designation (Iredale 1929). Subsequently, Barry (1993) characterised the genus as "turbinate, spirally ribbed, carinate; umbilicus wide and funnel-shaped, bordered by a nodulose funicle; and columella arched, simple, meeting the parietal wall at a steep angle". Although *Monilea* Swainson, 1840 and *Conotalopia* Iredale, 1929 are morphologically similar, the key difference is the carination on *Conotalopia*'s shoulders (Barry 1993). Information on the research of *Conotalopia* is rather limited. For certain Japanese taxa, descriptions of the shell and radula are available (Sowerby 1903, Taki and Oyama 1954, Oyama 1973, Okutani 2017).

According to the WoRMS, *Conotalopia* comprises only eight recent species, mostly in the Pacific Region (Gould 1861, Sowerby 1914). The genus has not previously been recorded in China. In 2020, the Laboratory of Shellfish Genetics and Breeding at the Ocean University of China conducted two field surveys in the Nanji Islands, China and identified several trochid-like gastropods with unique shell characteristics, such as *Conotalopiasematensis* (Oyama, 1942). To our knowledge, this is the first report of this genus in China.

## Materials and methods

Samples were collected from intertidal rocks covered with algae on Dalei Island (27°29.82'N, 121°06.17'E), Zhejiang, China, in July 2020. All specimens were preserved in 95% ethanol and stored at the Laboratory of Shellfish Genetics and Breeding (LSGB), Fisheries College, Ocean University of China, Qingdao, China. Before being selected for further analysis, the specimens were cleaned using ultrasound at 40 kHz for 30 s.

Standard views (top view, lateral view and bottom view) of the shells were captured using a DS-Fi2 digital camera (Nikon) mounted on a stereomicroscope. For scanning electron microscopy (SEM), radulae were collected during DNA extraction following the method outlined by Qi et al. (2020). The radulae were cleaned using 10% sodium hydroxide (NaOH) for 0.5 h and then rinsed with double-distilled water (ddH_2_O). The shells, radulae and opercula were mounted on stubs, thinly coated with gold and examined using a FlexSem 1000 II SEM.

Total genomic DNA was extracted from whole animals using the TIANamp Marine Animals DNA Kit (Tiangen Biotech, Beijing, China) following the manufacturer’s protocol and stored at 4°C for short-term use. The mitochondrial marker cytochrome oxidase subunit I (COI) was then amplified with the primers LCO1490 (GGTCAACAAATCATAAAGATATTGG) and HCO2198 (TAAACTTCAGGGTGACCAAAAAATCA) (Folmer et al. 1994). Each PCR sample (10 μl) contained 4 μl of DNA extract and 6 μl of PCR mix (0.2 μl ddH_2_O, 5 μl of 2× Taq Plus Master Mix II (Dye Plus; Vazyme, Nanjing, China), 0.4 μl of 10 μM forward primer and 0.4 μl of 10 μM reverse primer). The PCR conditions were as follows: a predenaturation at 94°C for 3 min; 35 cycles of denaturing at 94°C for 45 s, annealing at 48°C for 45 s and extension at 72°C for 60 s; and a final extension step at 72°C for 10 min. PCR products were confirmed by 1.5% agarose gel electrophoresis, stained with ethidium bromide, purified using the EZ Spin Column PCR Product Purification Kit (Sangon) and sequenced in both directions using an ABI 3730 automatic sequencer (Applied Biosystems) at the LiuHe HuaDa Biotechnology Company (Beijing, China). The sequences were assembled and manually curated using SeqMan (www.DNASTAR.com). Relevant COI sequences of the trochids were retrieved from GenBank (Suppl. material 1). Pairwise genetic distances (p‐distance) between datasets (Minolia, Conotalopia and Solariella) were calculated using MEGA 5 (Tamura et al. 2011).

## Taxon treatments

### Conotalopia sematensis

Oyama, 1942

5287E2B6-7C76-5002-BAD5-D9F7E4694F23

#### Materials

- **Type status:** Other material. **Occurrence:** catalogNumber: LSGB 20200608; recordedBy: Qi Lu; individualCount: 10; lifeStage: subadult; occurrenceID: 0D2C619C-7A59-56F1-816F-88E901CFB3B5; **Taxon:** scientificName: *Conotalopiasematensis*; kingdom: Animalia; phylum: Mollusca; class: Gastropoda; order: Vetigastropoda; family: Trochidae; genus: Conotalopia; specificEpithet: sematensis; taxonRank: species; scientificNameAuthorship: (Oyama, 1942); taxonomicStatus: accepted; **Location:** islandGroup: Nanji; island: Dalei; country: China; stateProvince: Zhejiang; locality: Nanji island National Nature Reserve, Dalei island; verbatimCoordinates: 27°29.82'N 121°06.17'E; georeferenceProtocol: label; **Identification:** identifiedBy: Qi Lu; dateIdentified: 2023
- **Type status:** Other material. **Occurrence:** lifeStage: adult; preparations: fossil; associatedReferences: https://www.biodiversitylibrary.org/item/101787#page/118/mode/1up, Yokoyama, M. (1922). Fossils from the Upper Musashino of Kazusa and Shimosa. Journal of the College of Science, Imperial University of Tokyo. 44(1): viii + 1-200.; occurrenceID: 504CC3F8-7EBC-5E9F-A5CF-127A977C3377; **Taxon:** scientificName: Solariella philippensis; kingdom: Animalia; phylum: Mollusca; class: Gastropoda; order: Vetigastropoda; family: Solariellidae; genus: Solariella; specificEpithet: philippensis; taxonRank: species; **Location:** country: Japan
- **Type status:** Other material. **Occurrence:** preparations: whole animal; associatedReferences: https://www.marinespecies.org/aphia.php?p=taxdetails&id=732128, Higo, S., Callomon, P. & Goto, Y. (1999) Catalogue and Bibliography of the Marine Shell-Bearing Mollusca of Japan. Elle Scientific Publications, Yao, Japan, 749 pp.; occurrenceID: AD459A05-88FA-5967-96F3-0F1AC508C0A4; **Taxon:** scientificName: Conotalopiasematensis; kingdom: Animalia; phylum: Mollusca; class: Gastropoda; order: Vetigastropoda; family: Trochidae; genus: Conotalopia; specificEpithet: sematensis; taxonRank: species; scientificNameAuthorship: (Oyama, 1942); taxonomicStatus: accepted; **Location:** country: Japan

#### Description

**Figure 1.**
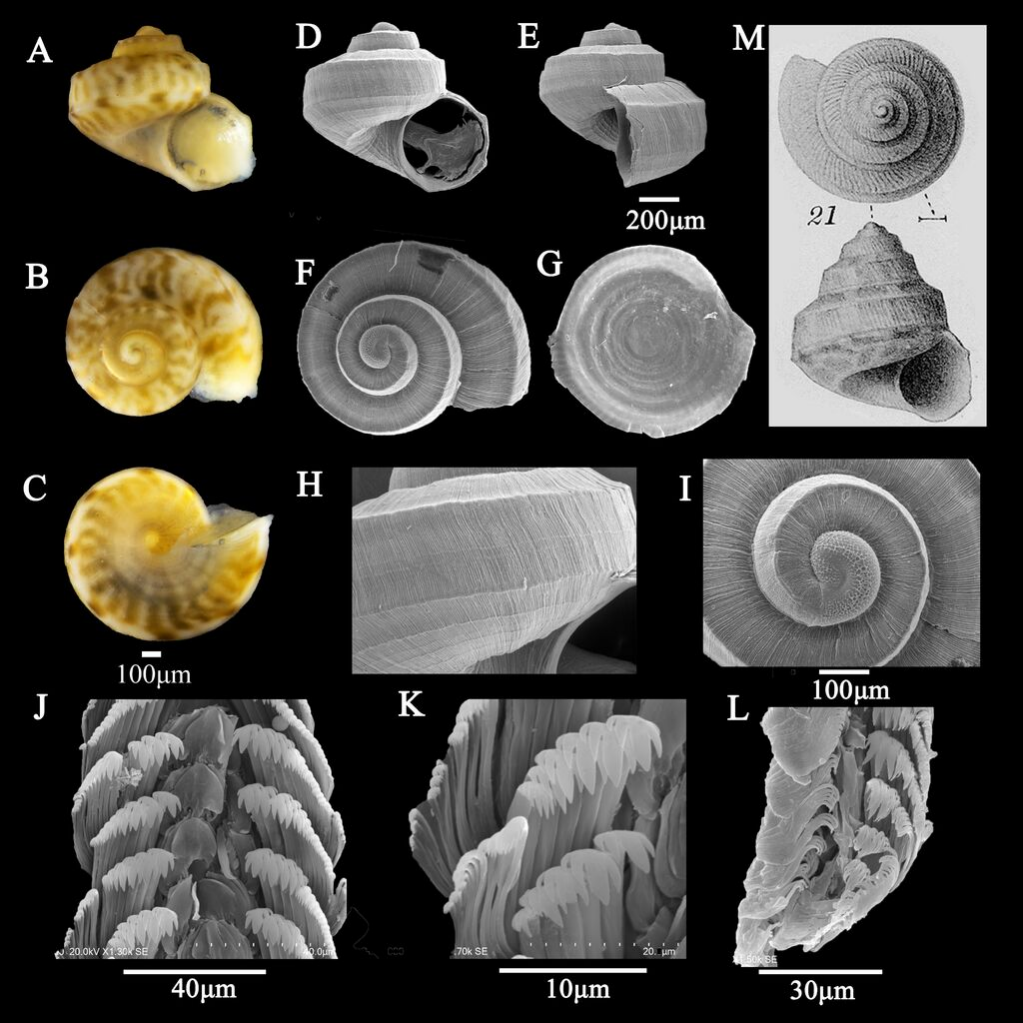
*Conotalopiasematensis* (Oyama, 1942). **A-C** Colour illustrations of shell; **D-F** Scanning electron micrographs of shell; **G** Operculum; **H** Surface of the shell; **I** Protoconch; **J-L** Radula; **M** Fossil shell of *C.sematensis* (Oyama, 1942) (Yokoyama 1922).

**Shell**: (Fig. 1A-E) small (1.5 mm ± 0.07 mm), low conical. Whorls exhibited irregular longitudinal bands alternating in colour between white and light yellow-brown or yellow-green (Fig. 1A-C). Three whorls, surface entirely covered with dense axial growth line; each whorl sharply angulated at shoulder, slightly sloped zone above the shoulder, below very steep, flat or slightly convex; the other angle was situated near the lower suture (Fig. 1D-F). Between these two angles, there were often one or two faint threads (Fig. 1H). Protoconch dextral; surface with reticular sculpture; clear boundary between protoconch and secondary shell (Fig. 1I). Suture obvious; five spiral ribs at the base. The umbilicus is open and deep; its margin is moderately angulated; and a cancellate sculpture is created by five spiral ribs and growth lines around the umbilicus. The aperture is simple and subcircular; the outer lip is thin with two corners; the inner lip is smooth; and one corner is at the bottom of the aperture.

**Operculum**: Horny, circular, yellowish, translucence, with a multispiral nucleus in the centre (Fig. 1G).

**Radula**: The radula type is rhipidoglossan radula (15+4+1+4+15) (Fig. 1J-L). The central teeth were spade-shaped, with smooth edges and no cusps. The lateral and marginal teeth formed closely-spaced rows at an angle of 45° on both sides of the central teeth in a symmetrical arrangement. Each row typically consisted of approximately four lateral teeth, with each lateral tooth having five small cusps (2+1+2). Additionally, each row typically included approximately 15 marginal teeth, with each marginal tooth featuring approximately seven small cusps (2-3+1+2-3). The apical teeth were the largest, with 2-3 denticles on each side.

#### Diagnosis

The shell surface was entirely covered by a dense axial growth line. Each whorl angulated at the shoulder, slightly carinated at the angle, slightly sloped above or below very steep, flat or slightly convex. There were often one or two fainter threads between the two angles.

#### Distribution

China (Zhejiang), Japan, Philippines, Australia.

## Analysis

The genetic distance between *C.sematensis* and the analysed species ranged from 16.1% to 22.6% (Table 1), indicating a close relationship with other *Conotalopia* species.

## Discussion

*Conotopala* differs from other trochids by turbinate, spirally ribbed, carinate, umbilicus wide and funnel-shaped, columella arched, simple and meeting the parietal wall at a steep angle, these characteristics also being observed in our samples. Our sample aligned with the description of *Conotalopia* provided by Barry (1993). Morphologically, our samples closely resembled *Conotalopiasematensis* (Oyama, 1942) (Fig. 1M), as documented by Yokoyama (1922), Yokoyama (1926), Taki and Oyama (1954) and Oyama (1973). The key diagnostic features that distinguish *C.sematensis* from other *Conotalopia* species include the presence of dense axial growth lines on the surface and one or two faint threads between the two angles. Although our specimens had a lower spire compared to the specimen described by Yokoyama (1922), the characteristics of the shell surface of our specimens are consistent with *Conotalopiasematensis* (Fig. 1M). The shell is sharply angulate a little above the middle, with a slightly sloping surface above the angle and a very steep surface below. There are one or two fainter threads on the surface of the whorls and oblique lines of growth can be seen everywhere. The periphery of the umbilicus is ornamented with spiral striae and crossed by lines of growth. These characteristics were observed in both our samples and *Conotalopiasematensis* (Fig. 1M). Therefore, we consider our specimens to be subadult.

Moreover, our small shells exhibited some similarities to *Conotalopiaornata* (G. B. Sowerby III, 1903), but detailed differences were evident. Notably, there was an absence of cancellated sculptures created by spiral ribs and growth lines on the surface of the whorls and the zone above the shoulders was slightly sloped rather than flat. In terms of the radula, the central tooth of *C.sematensis* is spade-shaped and the lateral teeth have cusps. In contrast, the central teeth of *C.ornata* have a somewhat protruding frontal margin, whereas the lateral teeth lack cusps (Habe 1958). Molecular studies support this distinction, with a genetic distance of 16.7% between our sample and *C.ornata* (Table 1). In summary, this species can be clearly distinguished from other *Conotalopia* species (Suppl. material 2).

## Supplementary Material

XML Treatment for Conotalopia sematensis

D3FC5346-FF74-5159-B205-25369D34D42D 10.3897/BDJ.12.e117114.suppl1 Supplementary material 1 GenBank accession numbers Data type Table Brief description GenBank accession numbers of specimens used for molecular analysis. File: oo_944728.docx https://binary.pensoft.net/file/944728 Lu Qi

A404B4A0-38C8-5895-9D62-7DE455F9C5D9 10.3897/BDJ.12.e117114.suppl2 Supplementary material 2 Comparison amongst *Conotalopia* species Data type Table Brief description A comparison amongst *Conotalopia* species. File: oo_942919.docx https://binary.pensoft.net/file/942919 Lu Qi

## Figures and Tables

**Table 1.** *P-distance* pairwise sequence distances (in percentage) between the analysed specimens based on the COI gene.

Species	1	2	3	4	5	6	7	8
***Conotalopiasematensis***								
*Conotalopiamustelina*	16.1							
*Conotalopiaornata*	16.7	15.8						
*Minoliachinensis*	18.5	15.5	18.1					
*Solariellanyssonus*	18.9	20.2	19.9	21.2				
*Minoliapunctata*	19.2	19.6	18.7	19.6	6.8			
*Minolia* sp.	19.3	19.5	18.5	19.6	6.6	0.2		
*Minoliasegersi*	22.6	20.6	22.7	22.1	17.3	16.7	16.7	
