# Supplementary material for: First record of the genus Conotalopia Iredale, 1929 (Vetigastropoda, Trochidae) in China
Source: Biodivers Data J. 2024 Jun 11;12:e117114. doi: 10.3897/BDJ.12.e117114 (PMC11188274; doi:10.3897/BDJ.12.e117114)
Supplement: Supplementary material 1 — GenBank accession numbers [file bdj-12-e117114-s001.docx]

| Species | GenBank Accession | Country |
| --- | --- | --- |
| *Solariella nyssonus* | HF586295.1 | Japan |
| *Conotalopia mustelina* | AB505302.1 | Japan |
| *Conotalopia ornata* | AB505303.1 | Japan |
| ***Conotalopia sematensis*** | OR839866 | China |
| *Minolia chinensis* | KP253008.1 | China |
| *Minolia punctata* | AB365226.1 | Japan |
| *Minolia segersi* | HF586323.1 | Philippines |
| *Minolia* sp. | HF586296.1 | Japan |

**Table 1**. Specimens analyzed in this study
