# Supplementary material for: First record of the genus Conotalopia Iredale, 1929 (Vetigastropoda, Trochidae) in China
Source: Biodivers Data J. 2024 Jun 11;12:e117114. doi: 10.3897/BDJ.12.e117114 (PMC11188274; doi:10.3897/BDJ.12.e117114)
Supplement: Supplementary material 2 — Comparison amongst Conotalopia species [file bdj-12-e117114-s002.docx]

**Suppl. file 1**

A comparison among *Conotalopia* species (“-”: no information available)

| **Species** | **Shell** | **Protoconch** | **Umbilicus** | **Aperture** | **Central teeth** | **Lateral teeth** | **Marginal teeth** | **Reference** |
| --- | --- | --- | --- | --- | --- | --- | --- | --- |
| *Conotalopia ornata* (G. B. Sowerby III, 1903) | Shell conical with high apex. Each whorl sharply angulate at two corners. Cancellate sculpture created by spiral ribs and growth lines in upper whorls, but weakened in lower whorls. |  | Umbilicus open; its margin moderately angulate. | Aperture subcircular |  |  |  | Okutani, 2016 |
| *Conotalopia henniana* (Melvill, 1891) | Whorls with sharply bicarinate; with a wide, nearly horizontal zone abrove the shoulder; concave sides | - | With a wide funnel-shaped entrance, |  |  |  |  | Barry, 1993 |
| *Conotalopia minima* (Golikov, 1967) | whorls carinated by two prominent spiral ribs. |  | Umbilicus not open. |  | - | - | - | Okutani, 2016  Sowerby, 1903 |
| *Conotalopia musiva* (A. Gould, 1861) | Body-whorl carinated by three prominent spriral ribs.  The upper part of spire has a smooth surface. |  | Umbilicus deep but narrow. |  | - | - | - | Gould, 1861 |
| *Conotalopia mustelina* (A. A. Gould, 1861) | Surface streaked with fine spiral threads. |  | Umbilicus narrow |  | - | - | - | Okutani, 2016 |
| *Conotalopia sematensis* (Oyama, 1942) | Whorls about five, sharply angulate a little above the middle. The sculpture consists generally of two spiral threads, the one forming the angle and the other situated near the lower suture. Between these two, there are often one or two fainter threads which sometimes may become rather coarse. oblique lines of growth are everywhere conspicuous | - | Umbilical margin bluntly angulate. Umbilical wall very steep and ornamented with spiral striæ crossed by lines of growth. | Aperture subcircular, with peristome discontinuous | - | - | - | Oyama, 1973; Taki, 1954; Yokoyama, 1922 |
| *Conotalopia singaporensis* (Pilsbry, 1889) | whorls about 5, very convex, those of spire bicarinate, the last whorl with two principal carinæ and several smaller ones on the base of the whorl, the entire surface very regularly strongly obliquely crispate-striate | - | umbilicus deep, funnel-shaped | aperture almost perfectly circular, in contact with the body-whorl for only a short distance, margins thin, outer and inner equally curved | - | - | - | Pilsbry, 1889 |
| *Conotalopia tropicalis* (Hedley, 1907) | Whorls angulate at the shoulder, centre and base; three strong spiral ribs encircle the centre of the whorls | - |  |  | - | - | - |  |
| *Conotalopia hilarula* (Yokoyama, 1926) | Whorls about four, convex with sutures more or less sunken, ornamented with five equally distributed spiral cords which on the body-whorl are faint and indistinct. Periphery quite rounded |  | Funnel-shaped, with several (about four) spiral grooves near its mouth. | Aperture subcircular, with peristome almost continuous. |  |  |  | Yokoyama, 1926 |

**Reference**

Gould A A (1861) Description of new shells collected by the United States North Pacific Exploring Expedition. Proceedings of the Boston Society of Natural 7: 385-389. https://www.biodiversitylibrary.org/page/9249945

Okutani T (2017) Marine mollusks in Japan. Tokai University Press, Tokyo, Japan, 771 pp.

Oyama K (1973) Revision of Matajiro Yokoyama's type Mollusca from the Tertiary and Quaternary of the Kanto area. Palaeontological Society of Japan, Japan, 148 pp.

Pilsbry H A (1889) Manual of conchology, structural and systematic, with illustrations of the species. Conchological Section, Academy of Natural Sciences, Philadelphia, 270 p.

Sowerby G B (1903) Descriptions of fourteen new species of marine molluscs from Japan. Annals and Magazine of Natural History 12: 496-501

Taki I, Oyama K (1954) Matajiro Yokoyama's the Pliocene and later faunas from the Kwanto region in Japan. Palaeontological Society of Japan, Japan, 68 pp.

Yokoyama M (1922) Fossils from the upper Musashino of Kazusa and Shimosa. Journal of the College of Science 44(1): 110.

Yokoyama M (1926) Fossil shells from Sado. In Yatsu N, Yamasaki N, Fujii K, Matsubara K, Goto S (Eds) Journal of the Faculty of Science, Imperial University of Tokyo. Section II, Geology, Mineralogy, Geography, Seismology. Tokyo University, Tokyo, 1(8): 249–312.
